# Supplementary figures and images for: In vivo antagonistic role of the Human T-Cell Leukemia Virus Type 1 regulatory proteins Tax and HBZ
Source: PLoS Pathog. 2021 Jan 20;17(1):e1009219. doi: 10.1371/journal.ppat.1009219 (PMC7817025; doi:10.1371/journal.ppat.1009219)

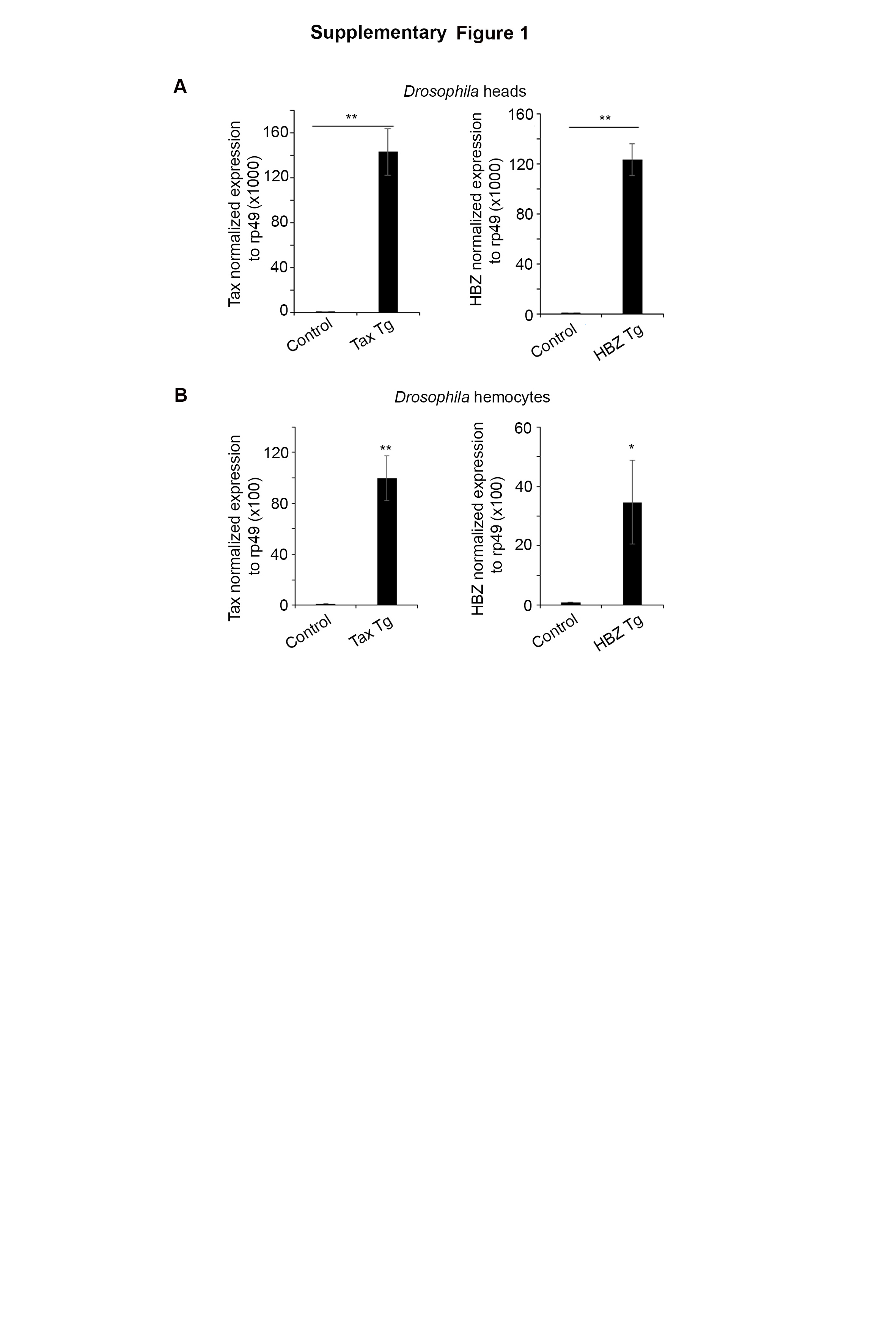

Supplement: S1 Fig — (A) Levels of expression of Tax and HBZ in the control (GMR-Gal4>w1118), Tax transgenics (Tg) (GMR-Gal4>UAS-Tax) and HBZ-Tg (GMR-Gal4>UAS-HBZ) confirming the expression of tax and hbz transgenes in adult flies heads. Transcript levels were normalized to Rp49. Reported values are the average of three independent experiments and error bars represent SD of triplicates. p<0.01 (**). (B) Levels of expression of Tax and HBZ in the control (HMLΔ-Gal4>w1118), Tax Tg (HMLΔ-Gal4>UAS-Tax) and HBZ-Tg (HMLΔ-Gal4>UAS-HBZ), confirming the expression of tax and hbz transgenes in larval hemocytes. Transcript levels were normalized to Rp49. Reported values are the average of three independent experiments and error bars represent SD of triplicates. p<0.1(*). (TIF) [file ppat.1009219.s001.tif]

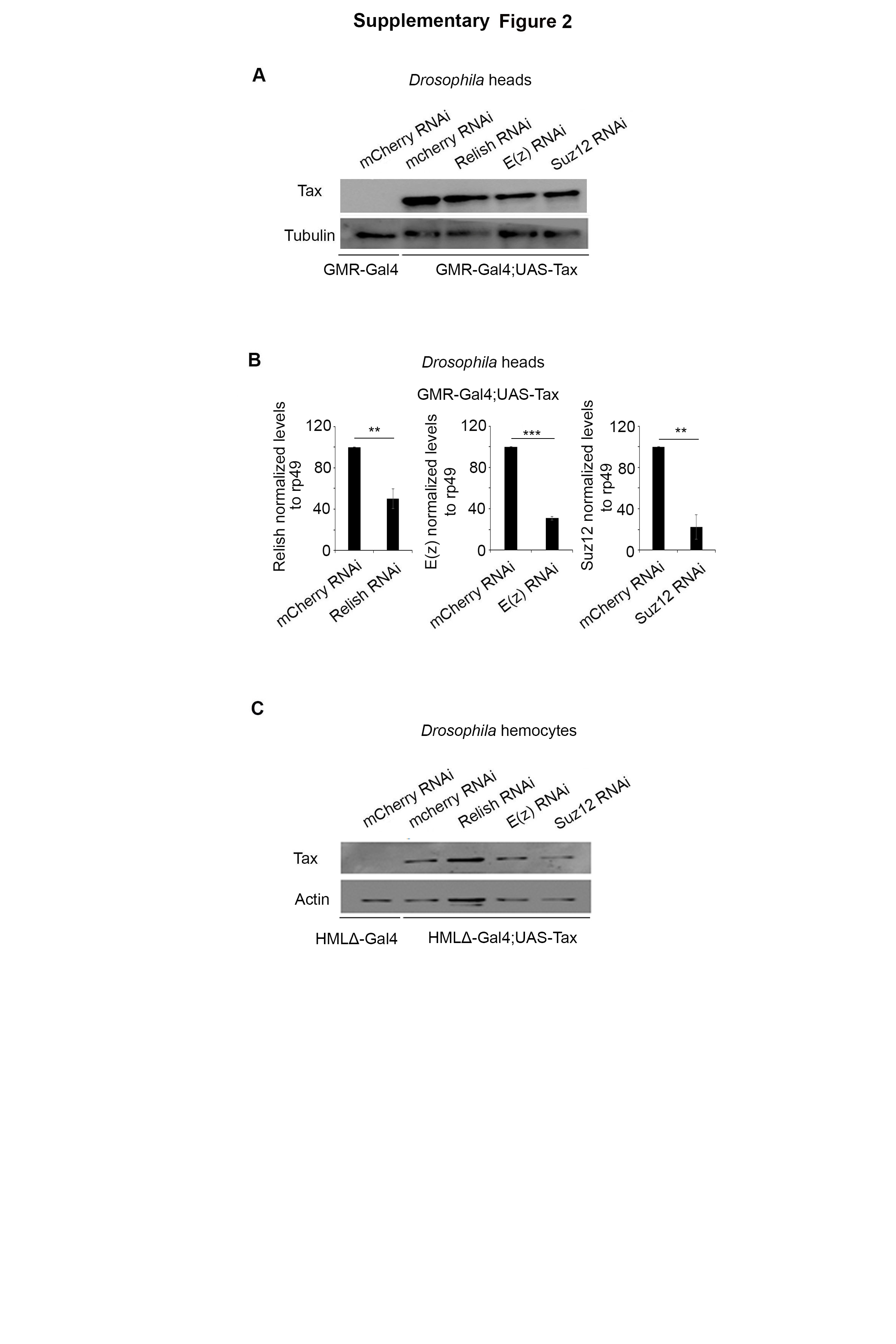

Supplement: S2 Fig — (A) Cell lysates (150 μg) of transgenic adult flies heads from control (GMR-Gal4>mCherry RNAi), (GMR-Gal4;UAS-Tax>mCherry RNAi), (GMR-Gal4;UAS-Tax>Relish RNAi), (GMR-Gal4;UAS-Tax>E(z) RNAi) and (GMR-Gal4;UAS-Tax>Suz12 RNAi) were analyzed by western blot confirming the expression of tax transgene. (B) Levels of expression of Relish, E(z), and SUZ12 in the transgenic adult flies heads as indicated. Transcript levels were normalized to Rp49. Reported values are the average of three independent experiments and error bars represent SD of triplicates. p<0.01 (**), p<0.001 (***). (C) Cell lysates (300 μg) from control (HMLΔ-Gal4> mCherry RNAi), (HMLΔ-Gal4;UAS-Tax>mCherry RNAi), (HMLΔ-Gal4;UAS Tax>Relish RNAi), (HMLΔ-Gal4;UAS-Tax>E(z) RNAi) and (HMLΔ-Gal4;UAS-Tax>Suz12 RNAi) transgenic larvae were analyzed by western blotting confirming the expression of Tax transgene in larval hemocytes. Indicated genotypes are under the control of the hemocyte-specific promoter (HMLΔ-GAL4). (TIF) [file ppat.1009219.s002.tif]

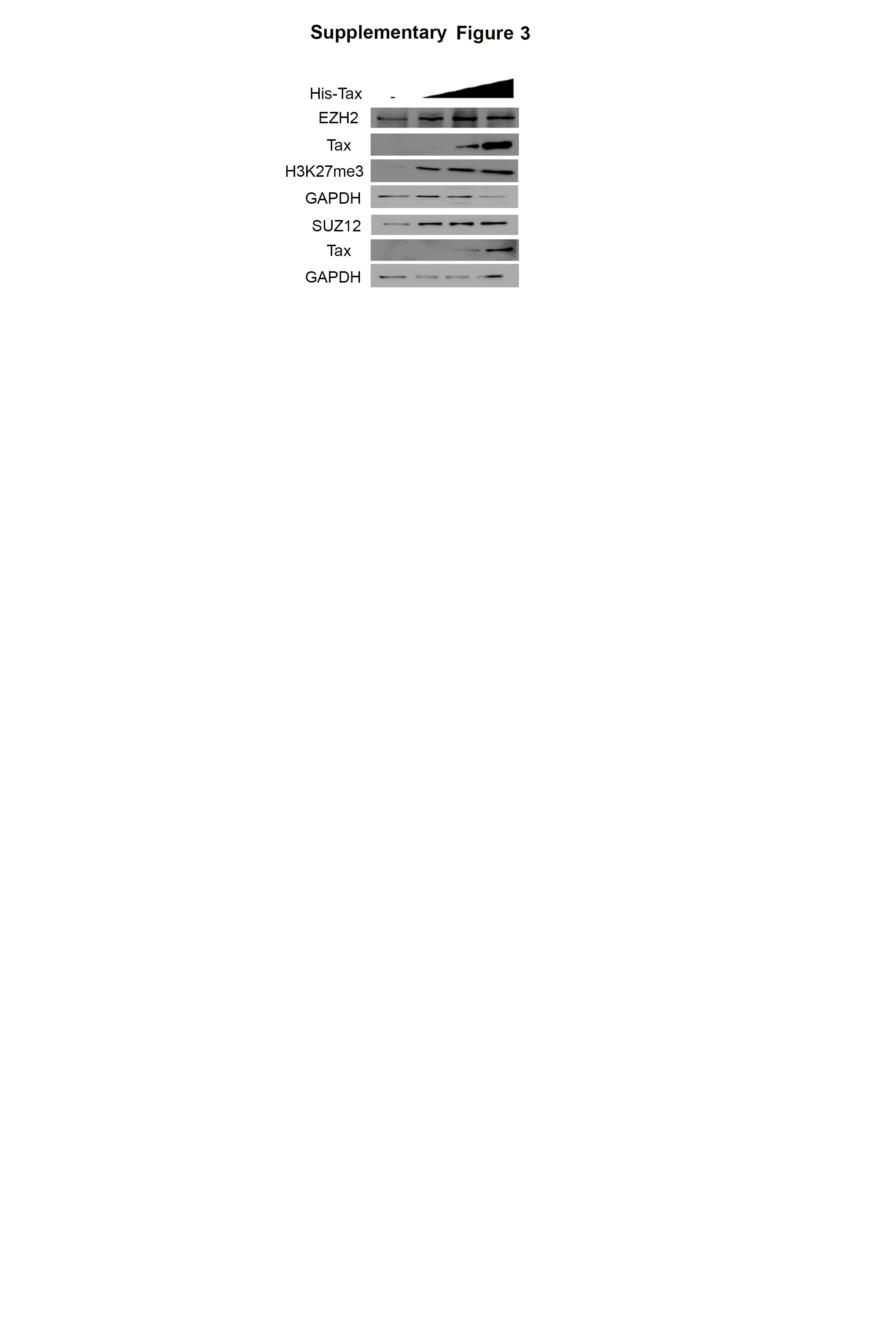

Supplement: S3 Fig — HEK293T cells were transiently transfected by His-Tax. Western blot was performed with indicated antibodies. (TIF) [file ppat.1009219.s003.tif]
