# Supplementary material for: In vivo antagonistic role of the Human T-Cell Leukemia Virus Type 1 regulatory proteins Tax and HBZ
Source: PLoS Pathog. 2021 Jan 20;17(1):e1009219. doi: 10.1371/journal.ppat.1009219 (PMC7817025; doi:10.1371/journal.ppat.1009219)
Supplement: S1 Table — (DOC) [file ppat.1009219.s004.doc]

**S1 Table.** List of PCR primers used in the study.

| Reaction | Target | Sequences |
| --- | --- | --- |
| Primers for RT-qPCR | Relish | Forward : 5′-CCACCAATATGCCATTGTGTGCCA-3′  Reverse : 5′-TTCCTCGACACAATTACGCTCCGT-3′ |
|  | Diptericin | Forward : 5′-ACTGCAAAGCCAAAACCATC-3′  Reverse : 5′-CCGCAGTACCCACTCAATCT-3′ |
|  | Tax | Forward : 5′-CGGATACCCAGTCTACGTGT-3′  Reverse : 5′-GAGCCGATAACGCGTCCATCG-3′ |
|  | HBZ | Forward : 5′-TAAACTTACCTAGACGGCGG-3′  Reverse : 5′- CTGCCGATCACGATGCGTTT-3′ |
|  | Dacapo | Forward : 5′-GCCCTTTAGCTGAAAATCACCC-3′  Reverse : 5′-GAGCCAAAGTTCTCCCGTTCT-3′ |
|  | d-jun | Forward : 5′-GCTAATTCCGCCGCCAATAA-3′  Reverse : 5′-CAATGGGATTAACGGTGGGC-3′ |
|  | GAPDH | Forward : 5′-GTGGACCTGACCTGCCGTCT-3′  Reverse : 5′-GGAGGAGTGGGTGTCGCTGT -3′ |
|  | Rp49 | Forward : 5′- CCGCTTCAAGGGACAGTATCTG-3′  Reverse : 5′- ATCTCGCCGCAGTAAACGC-3′ |
|  | CDKN1A | Forward : 5′- CTG TCA CTG TCT TGT ACC CTT GTG-3′  Reverse :5′-CGG CGT TTG GAG TGG TAG A-3′ |
|  | NDRG2 | Forward : 5'-AGC TGA CCG AGG CCT TCA AG-3'  Reverse :5'-CAA CGG ATG CTG CAC TGG TC-3' |
|  | BIM | Forward : 5'-CGG CGT ATT GGA GAC GAG TT-3'  Reverse : 5'-ACC ATT CGT GGG TGG TCT TC-3' |
| Primers for ChIP-qPCR: | CDKN1A | Forward : 5′-GGG GCG GTT GTA TAT CAG G-3′  Reverse : 5′-CTC TCT CAC CTC CTC TGA GTG C-3′ |
|  | NDRG2 | Forward : 5′-CAA AGG GCC CTA GAA TCT GTA TGT-3′  Reverse : 5′-GTT TCC CAC CCT TCT CAA GTG G-3′ |
|  | HEG1 | Forward : 5'-TGT CCT CGC GGT GAC ATC TC-3'  Reverse : 5'-ACG CCC TCT CAA GCT TGG AT-3' |
|  | GAPDH | Forward : 55′-AAC TTT CCC GCC TCT CAG C-3′  Reverse : 5′-CAG GAG GAC TTT GGG AAC GA-3′ |
|  | α-Satellite | Forward : 5′-CTG CAC TAC CTG AAG AGG AC  Reverse : 5′-GAT GGT TCA ACA CTC TTA CA-3′ |
|  | BIM | Forward : 5′-ACA ACG CCT CCT CAC TTG CT-3’  Reverse : 5'-AGG GAC CAC CCT ACA CAC CA-3' |
