# Supplementary material for: In vivo antagonistic role of the Human T-Cell Leukemia Virus Type 1 regulatory proteins Tax and HBZ
Source: PLoS Pathog. 2021 Jan 20;17(1):e1009219. doi: 10.1371/journal.ppat.1009219 (PMC7817025; doi:10.1371/journal.ppat.1009219)
Supplement: Attachment — Submitted filename: Response to reviewers (4).docx [file ppat.1009219.s005.docx]

October 30, 2020

Dear Editors

Thank you for your decision letter related to our manuscript **" *In vivo* antagonistic role of the Human T-Cell Leukemia Virus Type 1 regulatory proteins Tax and HBZ in oncogenic transformation"** submitted to PLOS Pathogens (no. PPATHOGENS-D-20-01735).

We have much appreciated the constructive comments from the reviewers and we would like to thank you for giving us the opportunity to resubmit it with the review. Please find on the following pages our point-by-point responses to the reviewers’ suggestions. We believe their comments significantly improved the revised manuscript. We will be happy to address any further comments or suggestions you or the reviewers might have.

With best regards,

On Behalf of the authors

Ali Bazarbachi, MD, PhD

**Point-by-point response**

**Editorial comments**

The reviewers agreed that your findings are of interest and potential importance. However, they also agreed that the manuscript as it stands has certain important limitations. Three points in particular need to be appropriately answered before the paper can be considered for publication.

First, you are asked to provide clearer and stronger justification for the use of the Drosophila model, which raises the fundamental question of how relevant the findings are to primates as the natural hosts of the virus.

We thank the editor and the reviewers for this important comment. We agree that ideally, mice models are potentially more relevant *in vivo* models than flies, to study the biology of HTLV-1, a virus known to infect primates. Indeed, different murine models of HTLV-1 infection or ATL were developed including transgenic, humanized or xenografted mice. These mice models were essential in understanding the pathogenesis of the disease and apprehending its potential therapeutics. Unfortunately, a main drawback in these murine models is the very long latency period, which exceeds 18 months for either *tax* transgenics ([Hasegawa et al.](https://www.ncbi.nlm.nih.gov/pmc/articles/PMC5882783/#B52) Nature medicine 2006), *hbz* transgenics ([Esser et al. Oncotarget. 2017](https://www.ncbi.nlm.nih.gov/pmc/articles/PMC5882783/#B30)) or *tax/hbz* double transgenics (Zhao et al. Archives of virology. 2014). This long latency period may be attributed to the acquisition of cellular somatic mutations, which makes it impossible to distinguish whether an observed phenotype or change in cell biology is attributed to the viral oncoprotein or to the acquired somatic mutations.

*Drosophila* emerged as a potent *in vivo* model for studying several diseases, including cancer, and especially the implication of epigenetics in cancer*.* Many advantages pertain to the use of *Drosophila* as a powerful *in vivo* tool in that setting. These include the fast generation time, the high number of progeny, and the availability of easy genetic screens. Indeed, a nearly complete collection of mutants, RNA interference (RNAi) lines, and a high number of driver systems (which allow for tangible expression of phenotypes in different sites including eyes and hemocytes), are accessible. In that sense, the eye in particular served as an *in vivo* tool that produces an easily identified “read-out” with the naked eye. Acquisition of a “rough-eye” phenotype is now an accepted surrogate for cell transformation in the *Drosophila* model. More importantly, key signaling pathways between mammals and flies are highly conserved including the NF-κB pathway (Hoffmann JA and Reichhart JM. Nature Immunology.2002) and the signaling pathways regulating blood cell differentiation. This clearly justifies the use of fruit fly models to study the basic mechanisms of hematopoietic differentiation and homeostasis and abnormalities responsible for severe diseases, including leukemia (Evans et al. Dev Cell. 2003; Tassetto et al. Cell. 2017). Finally, polycomb group proteins were originally discovered in *Drosophila* where their mutations resulted in improper body plans (Margueron R and Reinberg D. Nature. 2011). In particular, the polycomb PRC2 complex share a protein core complex that is conserved between Human (EZH1/2, SUZ12, EED, RBBP4/7) and *Drosophila* (E(z), Suz(12), Esc/EScI, Nurf55/Caf1) (Schuettengruber et al. Cell. 2017). For all these reasons, transgenic flies represent a powerful tool to study the *in vivo* properties of Tax and HBZ in terms of epigenetic changes, NF-κB activation, cellular proliferation and senescence. As such, most of the *in vivo* experiments would not have been reasonably feasible in a mouse model. Finally, many of our *in vivo* findings in transgenic flies were validated in transfected human cells and in ATL-derived cells (Figures 5, 6, supplementary Figure 3). The introduction and discussion of the revised manuscript are now modified, providing a justification of the use of *Drosophila* as an *in vivo* model.

Second, please explain how you reconcile the apparently conflicting actions of HBZ on NF-kappaB and PRC2, either activation or, in the presence of Tax protein, inhibition*.*

Regarding NF-κB, HBZ did not activate it neither alone nor in the presence of Tax. Indeed, HBZ reversed Tax-induced NF-κB activation. Regarding PRC2, based on our results, our hypothesis is that HBZ alone activates PRC2 activity, likely through upregulation of SUZ12 (Figures 1G and 6A) and direct interaction with EZH2 (Figure 6B and 6C). Conversely, HBZ suppresses Tax-induced PRC2 activation, likely because of inhibition of Tax-induced NF-κB activation (Figure 3E and 3F), resulting in inhibition of Tax-induced upregulation of EZH2 protein levels (Figure 3G,5A and 5B) as well as possibly through competitive binding of Tax and HBZ with components of the PRC2 complex (Figure 5G). This has been now added to the discussion and the model (Figure 7).

Third, two reviewers point out that Tax protein expression is lost in about 50% of cases of adult T-cell leukemia: this observation requires modification of some statements in the text of the paper on the roles of both Tax and HBZ, especially in leukemogenesis.

We thank the editor and the reviewers for this comment. Indeed, Tax expression in ATL cells was always debatable and is a controversial topic in the field. In that sense, in the original version of our manuscript, we clearly highlighted in the introduction “the undetectable Tax protein levels in most circulating HTLV-1 infected or ATL leukemic cells”.

We recently explored Tax expression in the PBMC from 6 patients with ATL (3 patients with acute ATL and 3 patients with chronic ATL). As control experiment, we performed serial dilutions of mRNA from the Tax expressing HuT-102 cell line with mRNA from the HTLV-1 negative CEM cell line. Comparing Tax transcript levels in primary PBMC from our six ATL patients to these serial dilutions of RNA of HuT-102 cells, we demonstrate that the level of expression of Tax transcripts in PBMC from acute ATL is 100 to 1000 times less than that of HuT-102 cells, while Tax expression in PBMC from chronic ATL patients is 50 to 100 times less than that of HuT-102 cells. These results clearly demonstrate that Tax is expressed in PBMC from ATL patients, but at much lower levels than cell lines. This low level of Tax expression in freshly isolated PBMC from ATL patients may originate from the leukemic cells or from the HTLV-1 infected non-malignant cells. These results are in preparation for another publication and are included here for reviewers only.

|  | mRNA TAX/GAPDH (log 10) |
| --- | --- |
| HuT102 | 2581.25 |
| 1:10 | 100 |
| 1:50 | 10.1532 |
| 1:100 | 6.81567 |
| 1:1000 | 2.51203 |
| Cem | 1.10869 |
| Acute P1 | 2.17929 |
| Acute P2 | 5.2193 |
| Acute P3 | 4.38889 |
| Chronic P1 | 10.8442 |
| Chronic P2 | 11.5824 |
| Chronic P3 | 11.1105 |

Moreover, the dependence of long-term survival of ATL-derived cells on Tax expression, is based on prior publications by our group and by others (Dassouki et al. Blood. 2015; Mahgoub et al. Proc Natl Acad Sci U S A. 2018), demonstrating that Tax knock-down in ATL-derived cells leads to cell death, even in cells with initially undetectable Tax expression. Transient bursts of Tax expression were indeed reported in ATL-derived cells and in HTLV-1 infected non-malignant cells (Billman et al. Wellcome Open Res. 2017; Mahgoub et al. Proc Natl Acad Sci U S A. 2018).

Nevertheless, and based on the reviewers comments, we have modified the discussion, in a more balanced way, as follows: “Although Tax protein is undetectable in most primary ATL cells, long-term survival of the bulk of ATL cells may depend on transient bursts of Tax expression in some, if not the majority, of ATL cells and/or in HTLV-1 infected non-malignant cells (Billman et al. Wellcome Open Res. 2017; Mahgoub et al. Proc Natl Acad Sci U S A. 2018). Conversely, HBZ is constantly expressed in almost all ATL cells suggesting a critical role for HBZ in the ATL phenotype”.

**REVIEWER #1**

**Part I - Summary**

Human T-cell leukemia virus type 1 (HTLV-1) encodes two viral oncoproteins, Tax and HBZ. In this article, Akkouche et al. show that HBZ inhibits the transforming activities of Tax in vivo using a transgenic Drosophila model. The authors previously reported that the Tax-transgenic Drosophila demonstrated several phenotypes associated with cellular transformation and NF-kappaB activation (Shirinian M. et al. JV, 2015). In the present study, they newly developed HBZ-transgenic Drosophila and a double transgenic strain of Tax and HBZ. Using those animal models, they show that both Tax and HBZ activate PRC2, resulting in the accumulation of H3K27me3 level. Interestingly, HBZ rather suppressed the activation of PRC2 and NF-kappaB, cellular transformation and senescence induced by Tax in their double transgenic flies. Finally, the authors conclude that HBZ and Tax play important roles in the persistence of HTLV-1.

**Part II – Major Issues**

1. In the transgenic flies, HBZ suppresses PRC2 activity only in the presence of Tax, although it seems that HBZ essentially has a property to activate PRC2. Mechanisms for those contradicting results have not been evaluated enough.

We thank the reviewer for this important comment. Based on our results, our hypothesis is that HBZ alone activates PRC2 activity, likely through upregulation of SUZ12 (Figures 1G and 6A) and direct interaction with EZH2 (Figure 6B and 6C). Conversely, HBZ suppresses Tax-induced PRC2 activation, likely because of inhibition of Tax-induced NF-κB activation (Figure 3E and 3F),), resulting in inhibition of Tax-induced upregulation of EZH2 protein levels (Figure 3G, 5A and 5B), as well as possibly through competitive binding of Tax and HBZ with components of the PRC2 complex (Figure 5G). This has been now added to the discussion and the model (Figure 7).

2. The authors suggest that Tax and HBZ competitively bind to EZH2 according to the result shown in Fig 5E. It is possible that Tax and HBZ target the same domain in EZH2 protein. If they can prove it, the result would strengthen their conclusion.

We thank the reviewer for this comment and we fully agree that this would be important to explore. Our results using immunoprecipitation and proximity ligation assay (Duolink®) suggest that both Tax and HBZ interact with EZH2 (Figure 5G, 6B and 6C). However, exploring the EZH2 domains involved in its interaction with Tax and with HBZ is reasonably beyond the scope of a revised manuscript. We have added that as future perspectives in the discussion.

3. As the authors mentioned in the manuscript, it has been reported that HBZ suppresses Tax-induced senescence by inibition of NF-kappaB hyperactivation, and promotes proliferation of Tax-expressing cells (Zhi H. et al. PLOS Pathog, 2011). Therefore, it is easy to understand the significance of NF-kappaB inactivation by HBZ for HTLV-1 persistence. On the other hand, there is almost no description about the meaning of PRC2 inhibition in Tax/HBZ co-expressing cells. How critical is PRC2 suppression by HBZ for Tax-expressing cells?

We thank the reviewer for this important comment. We reported in our originally submitted version that co-expression of Tax and HBZ in *Drosophila* rescued eye roughness, and decreased hemocyte count and senescence as compared to Tax transgenic (Tg) flies. Moreover, HBZ expression in Tax Tg flies partially decreased relish and diptericin levels, as compared to Tax alone, reflecting reversal of Tax-mediated NF-κB activation. To address the importance of the PRC2 complex in HBZ-mediated inhibition of Tax-induced proliferation, NF-κB activation and senescence, we knocked-down the expression of PRC2 core components, namely E(z) and SUZ12, in Tax Tg flies. We thus generated different fly lines: Tax/Relish RNAi (defective NF-κB pathway), Tax/E(z) RNAi and Tax/SUZ12 RNAi (defective PRC2 complex), and used Tax/mcherry RNAi as a control (Figure 2). As compared to the control Tax/mcherry RNAi line which exhibited eye roughness, increased hemocyte count, NF-κB activation and senescence, knock down of Relish totally reverted these Tax effects and significantly decreased E(z) and SUZ12 transcript levels (Figure 2H). Conversely, knock down of E(z) or SUZ12 only partially reverted Tax-induced eye roughness (Figure 2A, B), hemocyte count (Figure 2C), NF-κB activation (Relish and diptericin transcripts) (Figure 2G) and senescence (Figure 4F). Altogether, these results implicate the PRC2 core components E(z) and SUZ12 in Tax-mediated cell proliferation, NF-κB activation and senescence. Similarly, Tax-induced NF-κB activation enhanced PRC2 activity hence creating an activation loop. Therefore, PRC2 inhibition in Tax/HBZ co-expressing cells contributes to the reversal of Tax effects. These results are now added to Figure 2 and Figure 4 and the text of the results and discussion sections was modified accordingly.

Moreover, in the originally submitted version of the manuscript, ChIP assay showed that co-expression of Tax and HBZ in 293T cells resulted in a reduction of the enrichment level of H3K27me3 at the promoter regions of CDKN1A, NDRG2 and HEG-1, known to be Tax-controlled genes (Fujikawa et al. Blood. 2016). We have now performed real-time PCR to assess the expression of CDKN1A and NDRG2 in Tax, HBZ or Tax/HBZ transfected cells. Consistent with the H3K27me3 enrichment obtained in the ChIP assay, we show that the transcript levels of CDKN1A and NDRG2 are lower in Tax expressing cells and their levels are restored upon co-expression of Tax and HBZ (Figure 5D) These results are now added to Figure 5 and the text was modified accordingly. We then examined the enrichment level of H3K27me3 at the promoter region of the HBZ controlled gene BIM (Tanaka-Nakanishi et al. Cancer research. 2014), and demonstrated a specific increase in HBZ expressing cells. This increase was sustained upon co-expression of Tax and HBZ (Figure 5C). Consistent with the H3K27me3 enrichment obtained in the ChIP assay, we show that the transcript levels of BIM gene are lower in HBZ and Tax/HBZ expressing cells (Figure 5D). These results are now added to Figure 5 and the text was modified accordingly. Altogether, these results show the specificity of the effect of HBZ on Tax-dependent genes in Tax/HBZ expressing cells.

**Part III – Minor Issues**

1. Author summary needs to be revised since the content is quite similar to the Abstract.

As requested, the author summary is now revised.

2. Page 5, line 109. It’s assumed that a word “dispensable” is wrong. “indispensable”?

Corrected.

3. The authors show that Tax activates both NF-kappaB and PRC2. However, the association between those pathways/molecules is not clear. Yamagishi et al. previously reported that overexpression of PRC2 components suppresses the expression of miR-31 which targets NIK, leading to activation of NF-kappaB pathway in ATL cells (Yamagishi M. et al. Cancer Cell, 2012). Are there any links between NF-kappaB and PRC2 in their Drosophila model?

We thank the reviewer for this comment. We now measured the mRNA levels of the PRC2 components E(z), Suz12 and the NF-κB component Relish by RT-qPCR in Tax Tg following knockdown of Relish (Tax/Relish RNAi), knockdown of E(z) (Tax/E(z) RNAi) or Suz12 (Tax/Suz12 RNAi). We show that NF-κB knockdown in Tax/Relish RNAi flies decreased E(z) and Suz12 mRNA levels (Figure 2H) and that PRC2 knockdown in Tax/E(z) RNAi flies and Tax/Suz12 RNAi flies decreased Relish and diptericin mRNA as compared to the Tax/mCherry RNAi control flies (Figure 2G). These results suggest the existence of a bidirectional activation loop between the NF-κB pathway and the PRC2 complex. Concurrent with these observed differences of transcription in either knockdown lines, a significant decrease in H3K27me3 level was observed in both Tax/Relish RNAi, Tax/E(z) RNAi and Tax/Suz12 RNAi flies confirming a link between NF-κB and the PRC2 complex (Figure 2I). These results are now added to Figure 2 and the text was modified accordingly.

In Fig 2H, it is shown that knockdown of E(z) and Suz12 inhibited the induction of Diptericin induced by Tax; however, it is still obscure what the result means. They should explain about it.

In *Drosophila*, the NF-κB family member Relish is activated following the IMD (immune deficiency) pathway activation. We used Diptericin as a downstream effector of the activation of NF-κB in flies. This result confirms the link between NF-κB and PRC2 complex as explained in the previous paragraph.

4. In Fig 3D, the number of hemocytes is increased in Tax Tg flies but not in Tax/HBZ Tg. On the other hand, Fig 4D and E show that most of Tax Tg hemocytes (~80%) are senescent, while Tax/HBZ Tg hemocytes seem to be normal. If HBZ cancels cellular senescence induced by Tax, it is expected that hemocytes in the double Tg flies are increased, as Zhi H. et al. reported (Zhi H. et al. PLOS Pathog, 2011). How the authors explain those counterintuitive results?

Basically, HBZ expression in Tax Tg flies inhibits Tax-induced activation of the NF-κB pathway and Tax-induced activation of the PRC2 complex, likely explaining the inhibition of both cellular proliferation and subsequent senescence. Indeed, NF-κB knockdown (Relish RNAi) or PRC2 knockdown (E(z) RNAi) in Tax Tg flies reproduced HBZ effects and also inhibited both cellular proliferation and senescence.

**REVIEWER #2**

**Part I – Summary**

The authors demonstrate that antagonistic role of the Human T-Cell Leukemia Virus Type 1 regulatory proteins Tax and HBZ in oncogenic transformation in vivo.
They showed the evidence that antagonistic role of Tax and HBZ in oncogenic transformation in a gene modified fly system. They also performed in vitro experiment to analyze underlying molecular mechanism.
The study contains some new findings about the molecular characterization HTLV-1 viral protein Tax and HBZ in fly system. However, it is little evidence to understand HTLV-1 pathogenesis in human, because there are several missing points between experimental result and evidence observed in ATL patients in previous studies.

**Part II – Major Issues**

1. The first question is why they used the fly system. There are some explanations, but they would not be enough to convince readers.

We thank the editor and the reviewers for this important comment. We agree that ideally, mice models are potentially more relevant *in vivo* models than flies, to study the biology of HTLV-1, a virus known to infect primates. Indeed, different murine models of HTLV-1 infection or ATL were developed including transgenic, humanized or xenografted mice. These mice models were essential in understanding the pathogenesis of the disease and apprehending its potential therapeutics. Unfortunately, a main drawback in these murine models is the very long latency period, which exceeds 18 months for either *tax* transgenics ([Hasegawa et al.](https://www.ncbi.nlm.nih.gov/pmc/articles/PMC5882783/#B52) Nature medicine 2006), *hbz* transgenics ([Esser et al. Oncotarget. 2017](https://www.ncbi.nlm.nih.gov/pmc/articles/PMC5882783/#B30)) or *tax/hbz* double transgenics (Zhao et al. Archives of virology. 2014). This long latency period may be attributed to the acquisition of cellular somatic mutations, which makes it impossible to distinguish whether an observed phenotype or change in cell biology is attributed to the viral oncoprotein or to the acquired somatic mutations.

*Drosophila* emerged as a potent *in vivo* model for studying several diseases, including cancer, and especially the implication of epigenetics in cancer*.* Many advantages pertain to the use of *Drosophila* as a powerful *in vivo* tool in that setting. These include the fast generation time, the high number of progeny, and the availability of easy genetic screens. Indeed, a nearly complete collection of mutants, RNA interference (RNAi) lines, and a high number of driver systems (which allow for tangible expression of phenotypes in different sites including eyes and hemocytes), are accessible. In that sense, the eye in particular served as an *in vivo* tool that produces an easily identified “read-out” with the naked eye. Acquisition of a “rough-eye” phenotype is now an accepted surrogate for cell transformation in the *Drosophila* model. More importantly, key signaling pathways between mammals and flies are highly conserved including the NF-κB pathway (Hoffmann JA and Reichhart JM. Nature Immunology.2002) and the signaling pathways regulating blood cell differentiation. This clearly justifies the use of fruit fly models to study the basic mechanisms of hematopoietic differentiation and homeostasis and abnormalities responsible for severe diseases, including leukemia (Evans et al. Dev Cell. 2003; Tassetto et al. Cell. 2017). Finally, polycomb group proteins were originally discovered in *Drosophila* where their mutations resulted in improper body plans (Margueron R and Reinberg D. Nature. 2011). In particular, the polycomb PRC2 complex share a protein core complex that is conserved between Human (EZH1/2, SUZ12, EED, RBBP4/7) and *Drosophila* (E(z), Suz(12), Esc/EScI, Nurf55/Caf1) (Schuettengruber et al. Cell.2017). For all these reasons, transgenic flies represent a powerful tool to study the *in vivo* properties of Tax and HBZ in terms of epigenetic changes, NF-κB activation, cellular proliferation and senescence. As such, most of the *in vivo* experiments would not have been reasonably feasible in a mouse model. Finally, many of our *in vivo* findings in transgenic flies were validated in transfected human cells and in ATL-derived cells (Figures 5, 6, supplementary Figure 3). The introduction and discussion of the revised manuscript are now modified, providing a justification of the use of *Drosophila* as an *in vivo* model.

2. It would be better to put appropriate introduction. They describes several previous evidence to understand the roles of Tax and HBZ in ATL leukemogenesis. **It is important to know the situation in fresh ATL cells.** Several previous reports demonstrated that Tax expression is frequently silenced but HBZ expression is maintained in ATL cells (doi: 10.1073/pnas.0507631103, doi: 10.1038/ng.3415). Also, there are several evidences about high frequency of defective provirus in 5' side of HTLV-1. Thus, tax is not transcribed in such ATL cells due to lack of template DNA. For example, it is misleading to describe "Critically, long-term survival of ATL-derived cells depends on Tax expression [20, 21]: line 80-81". Because that may be true for some ATL cells, but we cannot generalize that.

We thank the reviewer for this comment. Indeed, Tax expression in ATL cells was always debatable and is a controversial topic in the field. In that sense, in the original version of our manuscript, we clearly highlighted in the introduction “the undetectable Tax protein levels in most circulating HTLV-1 infected or ATL leukemic cells”.

We recently explored Tax expression in the PBMC from 6 patients with ATL (3 patients with acute ATL and 3 patients with chronic ATL). As control experiment, we performed serial dilutions of mRNA from the Tax expressing HuT-102 cell line with mRNA from the HTLV-1 negative CEM cell line. Comparing Tax transcript levels in primary PBMC from our six ATL patients to these serial dilutions of RNA of HuT-102 cells, we demonstrate that the level of expression of Tax transcripts in PBMC from acute ATL is 100 to 1000 times less than that of HuT-102 cells, while Tax expression in PBMC from chronic ATL patients is 50 to 100 times less than that of HuT-102 cells. These results clearly demonstrate that Tax is expressed in PBMC from ATL patients, but at much lower levels than cell lines. This low level of Tax expression in freshly isolated PBMC from ATL patients may originate from the leukemic cells or from the HTLV-1 infected non-malignant cells. These results are in preparation for another publication and are included here for reviewers only.

|  | mRNA TAX/GAPDH (log 10) |
| --- | --- |
| HuT102 | 2581.25 |
| 1:10 | 100 |
| 1:50 | 10.1532 |
| 1:100 | 6.81567 |
| 1:1000 | 2.51203 |
| Cem | 1.10869 |
| Acute P1 | 2.17929 |
| Acute P2 | 5.2193 |
| Acute P3 | 4.38889 |
| Chronic P1 | 10.8442 |
| Chronic P2 | 11.5824 |
| Chronic P3 | 11.1105 |

Moreover, the dependence of long-term survival of ATL-derived cells on Tax expression, is based on prior publications by our group and by others (Dassouki et al. Blood. 2015; Mahgoub et al. Proc Natl Acad Sci U S A. 2018), demonstrating that Tax knock-down in ATL-derived cells leads to cell death, even in cells with undetectable Tax expression at baseline, such as MT1 cells. Transient bursts of Tax expression were indeed reported in ATL-derived cells and in HTLV-1 infected non-malignant cells (Billman et al. Wellcome Open Res. 2017; Mahgoub et al. Proc Natl Acad Sci U S A. 2018).

Nevertheless, and based on the reviewer comments, we have modified the discussion in a more balanced manner as follows: “Although Tax protein is undetectable in most primary ATL cells, long-term survival of the bulk of ATL cells may depend on transient bursts of Tax expression in some, if not the majority, of ATL cells and/or in HTLV-1 infected non-malignant cells (Billman et al. Wellcome Open Res. 2017; Mahgoub et al. Proc Natl Acad Sci U S A. 2018). Conversely, HBZ is constantly expressed in almost all ATL cells suggesting a critical role for HBZ in the ATL phenotype”.

3. Regarding role of NF-κB in ATL leukemogenesis.
NF-κB is not the focus of this study, but authors describes about the point to emphasise the role of Tax in ATL lekemogenesis.
There are some logical flaws. For example. not only viral factors but also host factors can explain activation of NF-κB in ATL cells. Actually a previous study demonstrated that high frequency of activation mutation in TCR signalling pathway (doi: 10.1038/ng.3415). Loss of mir31 expression can activate NF-κB signalling pathway in ATL cells (doi:10.1016/j.ccr.2011.12.015). It's fair to include these key papers when we discuss about NF-κB and ATL cells. Again, NF-κB is not main topic of this study.

We thank the reviewer for this comment and we agree that NF-κB is not the focus of this study. All suggested references are now added to the discussion of the revised manuscript as requested.

4. One of the key points about HTLV-1-mediated oncogenesis is cell-type specificity. HTLV-1 infects various cell types as well as CD4 T cells, but transform CD4 T cells almost exclusively. To address the points "role of the Human T-Cell Leukemia Virus Type 1 regulatory proteins Tax and HBZ in oncogenic transformation in vivo", I'd like to suggest authors to express viral genes in the CD4 T cells.

We thank the reviewer for this comment. In our approach, we used the GMR Gal4 promoter to specifically induce Tax, HBZ or Tax/HBZ expression in the Drosophila eyes and ensure a rapid read-out of the transformation capacity of either viral protein as well as of their *in vivo* effects on the PRC2 complex. To rule out any doubt related to the specificity of viral proteins to blood cells, we took advantage of the high conservation of the hematopoietic system, its development and differentiation processes, between humans and *Drosophila* as well as the ease of changing promoter to analyze the effect of cell type specificity upon expressing Tax, HBZ or both. We used the hemocyte specific promoter hemolectin (HMLΔ-Gal4) to direct the expression of Tax, HBZ or Tax/HBZ to *Drosophila* circulating hemocytes.

Due to the lack of CD4 T cells in the *Drosophila* hematopoietic system and to address the reviewer’s suggestion, we electroporated the human CD4^+^-T-cell line, Jurkat, with either an empty vector (control), His-Tax alone, Myc-HBZ alone, or with His-Tax/Myc-HBZ vectors, and assessed the downstream H3K27me3 by Western blot. Consistent with the fly and co-transfection experiments in 293T cells, overexpression of Tax alone or HBZ alone yielded an increase in SUZ12 and H3K27me3, while only Tax increased EZH2 (Figure 5B). Importantly, co-expression of both Tax and HBZ decreased the global amount of H3K27me3 as compared to Jurkat cells expressing either viral proteins (Figure 5B). A decrease in EZH2 and SUZ12 was also observed as compared to Jurkat cells expressing Tax alone, explaining the observed decrease in H3K27me3 (Figure 5B). These results confirm our findings in a CD4 T cell system. These results are now included in Figure 5 and the text was modified accordingly.

**REVIEWER #3**

**Part I – Summary**

In the present study, Akkouche et al. investigate the functional role of HTLV-1 HBZ and its interaction with the Tax oncoprotein using their transgenic Drosophila model. The rough eye phenotype and increased hemocyte count are used as indicators of transformation. Results in this model are in part validated using in vitro overexpression experiments in HEK cells and HBZ silencing experiments in the MT-1 cell line.
Results show that, like Tax, HBZ is capable of enhancing activity of the PRC complex, resulting in increased H3K27me3 repressive marks. Unlike Tax, however, HBZ neither induces “transformation”, nor NF-κB activation and instead antagonizes the positive effects of Tax on cellular transformation and NF-kB activation in the Drosophila model.
The paper is clearly written and the experimental setup well laid-out. Although the experiments are well-executed and the results are clear, there is some concern as to how much information can really be gained from the Drosophila model, when many transgenic mouse models for Tax and HBZ, as well as humanized mouse models are available. To this effect, it is puzzling that HBZ alone is oncogenic in mice while it clearly is not in Drosophila*.*

We thank the reviewer for this important comment. We agree that ideally, mice models are potentially more relevant *in vivo* models than flies, to study the biology of HTLV-1, a virus known to infect primates. Indeed, different murine models of HTLV-1 infection or ATL were developed including transgenic, humanized or xenografted mice. These mice models were essential in understanding the pathogenesis of the disease and apprehending its potential therapeutics. Unfortunately, a main drawback in these murine models is the very long latency period, which exceeds 18 months for either *tax* transgenics ([Hasegawa et al.](https://www.ncbi.nlm.nih.gov/pmc/articles/PMC5882783/#B52) Nature medicine 2006), *hbz* transgenics ([Esser et al. Oncotarget. 2017](https://www.ncbi.nlm.nih.gov/pmc/articles/PMC5882783/#B30)) or *tax/hbz* double transgenics (Zhao et al. Archives of virology. 2014). This long latency period may be attributed to the acquisition of cellular somatic mutations, which makes it impossible to distinguish whether an observed phenotype or change in cell biology is attributed to the viral oncoprotein or to the acquired somatic mutations.

*Drosophila* emerged as a potent *in vivo* model for studying several diseases, including cancer, and especially the implication of epigenetics in cancer*.* Many advantages pertain to the use of *Drosophila* as a powerful *in vivo* tool in that setting. These include the fast generation time, the high number of progeny, and the availability of easy genetic screens. Indeed, a nearly complete collection of mutants, RNA interference (RNAi) lines, and a high number of driver systems (which allow for tangible expression of phenotypes in different sites including eyes and hemocytes), are accessible. In that sense, the eye in particular served as an *in vivo* tool that produces an easily identified “read-out” with the naked eye. Acquisition of a “rough-eye” phenotype is now an accepted surrogate for cell transformation in the *Drosophila* model. More importantly, key signaling pathways between mammals and flies are highly conserved including the NF-κB pathway (Hoffmann JA and Reichhart JM. Nature Immunology.2002) and the signaling pathways regulating blood cell differentiation. This clearly justifies the use of fruit fly models to study the basic mechanisms of hematopoietic differentiation and homeostasis and abnormalities responsible for severe diseases, including leukemia (Evans et al. Dev Cell. 2003; Tassetto et al. Cell. 2017). Finally, polycomb group proteins were originally discovered in *Drosophila* where their mutations resulted in improper body plans (Margueron R and Reinberg D. Nature. 2011). In particular, the polycomb PRC2 complex share a protein core complex that is conserved between Human (EZH1/2, SUZ12, EED, RBBP4/7) and *Drosophila* (E(z), Suz(12), Esc/EScI, Nurf55/Caf1) (Schuettengruber et al. Cell.2017). For all these reasons, transgenic flies represent a powerful tool to study the *in vivo* properties of Tax and HBZ in terms of epigenetic changes, NF-κB activation, cellular proliferation and senescence. As such, most of the *in vivo* experiments would not have been reasonably feasible in a mouse model. Finally, many of our *in vivo* findings in transgenic flies were validated in transfected human cells and in ATL-derived cells (Figures 5, 6, supplementary Figure 3). The introduction and discussion of the revised manuscript are now modified, providing a justification of the use of *Drosophila* as an *in vivo* model.

One must also consider that in > 50% of ATL Tax expression is lost due to 5’ deletions spanning the x-IV ORF or to 5’LTR methylation, while HBZ expression is conserved, suggesting that ATL cells are addicted to HBZ (but not Tax) expression and that, in the context of leukemia cells, HBZ function is likely to be distinct from its capacity to overcome Tax-induced senescence. This suggests that, at least in a significant proportion of leukemia cells, “alleviating the deleterious effects” of the transient bursts of Tax expression is not a likely explanation of HBZ function and is not sufficient to reconcile the apparently contrasting results obtained in the fly model.

We fully agree with the reviewer that HBZ function is not limited to its capacity to overcome Tax-induced senescence in leukemia cells. We apologize if that was the impression that was given in our original manuscript. Our findings indicate that in Tax expressing cells, HBZ expression inhibits Tax-induced PRC2 activation, NF-κB activation, cell proliferation and senescence. We also show that in cells that are not expressing Tax, HBZ activates the PRC2 complex leading to H3K27me3 of HBZ dependent genes. These effects may not be restricted to leukemic cells but are likely to be the same in HTLV-1 infected non-leukemic cells. Transient bursts of Tax expression were indeed reported in ATL-derived cells and in HTLV-1 infected non-malignant cells (Billman, et al., Wellcome Open Res. 2017; Mahgoub et al. Proc Natl Acad Sci U S A. 2018). We speculate that these transient bursts of Tax expression are necessary for long-term self-renewal but that long-term Tax expression can lead to senescence or elimination of Tax expressing cells by the immune system. We also speculate that outside these transient bursts of Tax expression, HBZ maintains the PRC2 activation and its mediated key cellular functions in HTLV-1 infected or ATL cells. This is now clarified in the discussion.

In summary, the paper would greatly benefit if the authors would make a stronger point of the advantage of using their Drosophila model, and make an effort to integrate their (partly puzzling) results in a working model*.*

The below working model is now added to the revised manuscript (Figure 7).

**References**

Hasegawa H, Sawa H, Lewis MJ, Orba Y, Sheehy N, Yamamoto Y, et al. Thymus-derived leukemia-lymphoma in mice transgenic for the Tax gene of human T-lymphotropic virus type I. Nature medicine. 2006;12(4):466-72.

Esser AK, Rauch DA, Xiang J, Harding JC, Kohart NA, Ross MH, et al. HTLV-1 viral oncogene HBZ induces osteolytic bone disease in transgenic mice. Oncotarget. 2017;8(41):69250-63.

Zhao T, Satou Y, Matsuoka M. Development of T cell lymphoma in HTLV-1 bZIP factor and Tax double transgenic mice. Archives of virology. 2014;159(7):1849-56.

Hoffmann JA, Reichhart JM. Drosophila innate immunity: an evolutionary perspective. Nature immunology. 2002;3(2):121-6.

Evans CJ, Hartenstein V, Banerjee U. Thicker than blood: conserved mechanisms in Drosophila and vertebrate hematopoiesis. Developmental cell. 2003;5(5):673-90.

Tassetto M, Kunitomi M, Andino R. Circulating Immune Cells Mediate a Systemic RNAi-Based Adaptive Antiviral Response in Drosophila. Cell. 2017;169(2):314-25.e13.

Schuettengruber B, Bourbon HM, Di Croce L, Cavalli G. Genome Regulation by Polycomb and Trithorax: 70 Years and Counting. Cell. 2017;171(1):34-57.

Dassouki Z, Sahin U, El Hajj H, Jollivet F, Kfoury Y, Lallemand-Breitenbach V, et al. ATL response to arsenic/interferon therapy is triggered by SUMO/PML/RNF4-dependent Tax degradation. Blood. 2015;125(3):474-82.

Mahgoub M, Yasunaga JI, Iwami S, Nakaoka S, Koizumi Y, Shimura K, et al. Sporadic on/off switching of HTLV-1 Tax expression is crucial to maintain the whole population of virus-induced leukemic cells. Proceedings of the National Academy of Sciences of the United States of America. 2018;115(6):E1269-e78.

Billman MR, Rueda D, Bangham CRM. Single-cell heterogeneity and cell-cycle-related viral gene bursts in the human leukaemia virus HTLV-1. Wellcome open research. 2017;2:87.

Tanaka-Nakanishi A, Yasunaga J, Takai K, Matsuoka M. HTLV-1 bZIP factor suppresses apoptosis by attenuating the function of FoxO3a and altering its localization. Cancer research. 2014;74(1):188-200.

Fujikawa D, Nakagawa S, Hori M, Kurokawa N, Soejima A, Nakano K, et al. Polycomb-dependent epigenetic landscape in adult T-cell leukemia. Blood. 2016;127(14):1790-802.

Margueron R, Reinberg D. The Polycomb complex PRC2 and its mark in life. Nature. 2011;469(7330):343-9.
