# Supplementary material for: In vivo antagonistic role of the Human T-Cell Leukemia Virus Type 1 regulatory proteins Tax and HBZ
Source: PLoS Pathog. 2021 Jan 20;17(1):e1009219. doi: 10.1371/journal.ppat.1009219 (PMC7817025; doi:10.1371/journal.ppat.1009219)
Supplement: Attachment — Submitted filename: Response to reviewers.docx [file ppat.1009219.s006.docx]

November 28, 2020

Dear Esteemed Editors

Thank you for the decision related to our manuscript **"*In vivo* antagonistic role of the Human T-Cell Leukemia Virus Type 1 regulatory proteins Tax and HBZ in oncogenic transformation"** submitted to PLOS Pathogens (no. PPATHOGENS-D-20-01735).

We have much appreciated that the reviewers found that we indeed addressed most of their raised concerns. We are delighted that what is still requested, although minor, is constructive to further improve the quality of our manuscript. Please find below our point-by-point responses to the reviewers’ suggestions.

With best regards,

On Behalf of the authors

Ali Bazarbachi, MD, PhD

**Point-by-point response**

**Editorial comments**

Reviewers 1 and 3 consider that the results are overinterpreted, because of the doubt over how justifiable it is to extend the results in Drosophila to the manifestly different system in the natural host. Please therefore modify the title and the discussion in line with the comments made by the reviewers, making a clearer distinction between the Drosophila and mammalian systems, and be more circumspect in drawing conclusions as to the pathogenesis in the natural host.

We thank the editorial board for these comments. We have now modified the title to “*In vivo* antagonistic role of the Human T-Cell Leukemia Virus Type 1 regulatory proteins Tax and HBZ” hence deleting “oncogenic transformation” that the reviewers found controversial.

We also modified the discussion and the conclusion, by making a clear difference between the non-mammalian Drosophila model and the mammalian systems.

- Discussion Line 372; we added: “Yet, these interesting *in vivo* findings in a *Drosophila* model still require further validation in mammalian systems”
- Conclusion Line 433; we attenuated the previous statement by changing it to: “In conclusion and based on the generated data in *Drosophila* transgenic models, we hypothesized that Tax and HBZ play important roles in HTLV-I transformation and persistence”
- Conclusion Line 442; we added: “Again, these results demonstrated in a fly system will require further validation in a mammalian cell system”.
- Conclusion Line 444; we attenuated the previous statement by changing it to: “Overall, and despite a number of limitations in a non-mammalian system, we report a powerful *in vivo* tool to investigate the antagonistic effects of HBZ on Tax-induced transformation, cellular effects and epigenetic modulation.”

**REVIEWER #1**

**Part I - Summary**

The authors responded to most of the questions.

We are delighted that reviewer 1 was satisfied with our manuscript revision.

**Part II – Major Issues**

None

**Part III – Minor Issues**

1. Fig 2I: There seems to be faint, but not clear, difference between Tax+/mCherry RNAi (lane 2) and Tax+/Relish RNAi lane 3). Since this result is important to show that PRC2 activity is correlated with NFkB in this animal model, quantification and statistical evaluation of H3K27me3 levels should be done as the authors showed in Fig 1G.

We thank the reviewer for this comment. The gel in figure 2I was now replaced by a better one. Densitometry along with statistical evaluation of H3K27me3 levels from three independent experiments were now added to panel 2I in Figure 2.

**REVIEWER #2**

**Part I – Summary**

The authors demonstrate that antagonistic role of the Human T-Cell Leukemia Virus Type 1 regulatory proteins Tax and HBZ in oncogenic transformation in vivo.
They showed the evidence that antagonistic role of Tax and HBZ in oncogenic transformation in a gene modified fly system. They also performed in vitro experiment to analyze underlying molecular mechanism.
The finding is really interesting, but there is not enough evidence to support the title "In vivo antagonistic role of the Human T-Cell Leukemia Virus Type 1 regulatory proteins Tax and HBZ in oncogenic transformation".

We are delighted that Reviewer 2 emphasized on the importance of our work. As per his/her request, the title of the revised manuscript is now modified to “*In vivo* antagonistic role of the Human T-Cell Leukemia Virus Type 1 regulatory proteins Tax and HBZ” hence deleting “oncogenic transformation” that the reviewer may have found controversial.

**Part II – Major Issues**

1. Justification for the use of the Drosophila model
There are additional experiments using human cells. Tax and/or HBZ is over-expressed in 293T or Jurkat cells. It would be more convincing if the authors would compare the expression level of them with that in vivo in their Drosophila model and primary ATL cells. In the Drosophila model, Tax and HBZ protein are detected by W.B as shown Fig1E. Especially Tax expression level seems to be high. Since we rarely see such protein expression level in vivo in mouse model and infected individuals.
It would be more convincing if the authors show that transgene expression level is close to physiological level in vivo.

We thank the reviewer for this comment. Indeed, in our study, we did not claim that the transgene expression of either viral protein is close to its physiological level *in vivo*. We agree with the reviewer that Tax protein is undetectable by Western blot in PBMCs from most HTLV-1 infected individuals or primary ATL cells. Yet, recent data showed that bursts of Tax occur in a very small percentage of infected cells at one time. In this study, we expressed the Tax transgene under strong promoters (GMR-Gal4 and HMLΔ-Gal4). Therefore, all transgenic cells are expressing Tax which becomes detectable by Western blot contrary to HTLV-1 infected individuals or primary ATL cells. Our hypothesis is that, under physiological levels in HTLV-1 infected individuals, there are infected cells that express Tax alone, others that express HBZ alone or both Tax and HBZ. Using the fly system, we tried to mimic each of these three situations and investigated PRC2 activation, NF-kB activity, senescence and proliferation capacity in each setting. Nevertheless, based on the reviewer comments, we have now revised our discussion and conclusion by clearly stating the difference between the non-mammalian and mammalian systems, as well as the advantages and the drawbacks of these models.

- Discussion Line 372; we added: “Yet, these interesting *in vivo* findings in a *Drosophila* model still require further validation in mammalian systems”
- Conclusion Line 433; we attenuated the previous statement by changing it to: “In conclusion and based on the generated data in *Drosophila* transgenic models, we hypothesized that Tax and HBZ play important roles in HTLV-I transformation and persistence”
- Conclusion Line 442; we added: “Again, these results demonstrated in a fly system will require further validation in a mammalian cell system”.
- Conclusion Line 444; we attenuated the previous statement by changing it to: “Overall, and despite a number of limitations in a non-mammalian system, we report a powerful *in vivo* tool to investigate the antagonistic effects of HBZ on Tax-induced transformation, cellular effects and epigenetic modulation.”

2. Viral gene expression in ATL cells.
I do appreciate the authors' effort to analyze PBMCs from 6 ATL cases. However, there is little information about the details of the ATL cases. What is the proportion of ATL cells and non ATL-HTLV-1 infected cells in the PBMCs. It is difficult to know whether the viral gene expression detected comes from ATL cells or non-malignant infected cells. Flow cytometry analysis will be useful to provide such information (doi: 10.1158/1078-0432.CCR-13-3169.).

We thank the reviewer for this comment and we fully agree with the suggested experiments. Indeed, we have additional preliminary data demonstrating that the majority of primary ATL cells are still capable of expressing Tax protein. As mentioned in the previous response to reviewers comments, these data are the subject of another publication in preparation, and are beyond the scope of this paper. Some of them were previously included as “for reviewer only”. We count on the fair assessment of the reviewer and the editorial board.

**REVIEWER #3**

**Part I – Summary**

Reviewer #3: The Authors have significantly improved the quality of their manuscript providing new data to address most of the criticisms raised for their previous submission.

We thank Reviewer 3 for his/her effort in reviewing our manuscript and we are glad that the revised manuscript met his/her expectations.
